# Primary Adrenal Insufficiency Caused by a Novel Mutation in DAX1 Gene

**DOI:** 10.4274/Jcrpe.895

**Published:** 2013-03-21

**Authors:** Olcay Evliyaoğlu, İpek Dokurel, Feride Bucak, Bahar Özcabı, Oya Ercan, Serdar Ceylaner

**Affiliations:** 1 İstanbul University Cerrahpaşa Faculty of Medicine, Department of Pediatric Endocrinology, İstanbul, Turkey; 2 Intergen, Genetic Diagnosis Research and Application Center, Ankara, Turkey

**Keywords:** adrenal, Development, insufficiency, DAX1, mutation

## Abstract

Adrenal hypoplasia congenita (AHC) is a rare disorder. The X-linked form is related to mutations in the DAX1 (NROB1) gene. Here, we report a newborn who had a novel hemizygous frameshift mutation in DAX1 (c.543delA) and presented with primary adrenal failure that was initially misdiagnosed as congenital adrenal hyperplasia. This report highlights the value of genetic testing for definite diagnosis in children with primary adrenal failure due to abnormal adrenal gland development, providing the possibility both for presymptomatic, and in cases with a sibling with this condition, for prenatal diagnosis.

**Conflict of interest:**None declared.

## INTRODUCTION

Primary adrenal insufficiency can be caused by a deficiency in steroid biosynthesis or an abnormal adrenal gland development. Adrenal hypoplasia congenita (AHC) is an inherited disorder of adrenal development resulting in primary adrenal insufficiency (1). It has an estimated frequency of 1:12 500 live births (2) and can be inherited as an autosomal recessive or an X-linked disease (3). ?X-linked AHC is caused by deletions or mutations in DAX1 gene (dosage-sensitive sex reversal, AHC critical region on the X chromosome, gene 1; NROB1) situated in Xp21 (4). The DAX1 gene consists of two exons of 1168 and 245 bp, respectively, and encodes a 470 amino acid protein which belongs to the nuclear hormone receptor superfamily (5). DAX1 is expressed in the adrenal cortex, in the pituitary, in the hypothalamic ventral nucleus, in Sertoli and Leydig cells in the testis, and in theca and granulosa cells in the ovary (3,5,6). X-linked AHC has variable clinical presentations (7,8,9). Boys with DAX1 mutations typically present with primary adrenal failure. In these patients, the adrenal gland fails to develop fully. Histologically, the adrenal cortex permanent zone is absent, and there are residual cytomegalic cells (10). Urgent mineralocorticoid and glucocorticoid replacement is mandatory (11). Most boys fail to enter puberty and must be given testosterone replacement therapy in adulthood.

Here, we report a male newborn with AHC who presented with adrenal insufficiency due to a novel mutation in the DAX1 gene.

## CASE REPORT

A 33-day-old male infant who presented with vomiting and failure to thrive was admitted to the intensive care unit (ICU). His physical examination revealed severe dehydration with normal anthropometric measurements [(length: 52 cm (3-10%), weight: 3150g (10%), head circumference: 37 cm (25-50%)], normal male genital development, and hyperpigmentation of the scrotum and nipples. Prenatal and birth history (term, birth weight: 3000 g) were unremarkable. Parents were fourth-degree relatives. Biochemical measurements showed hyponatremia (Na: 123 mmol/L), hypochloremia (Cl: 79 mmol/L), hyperkalemia (K: 8.9 mmol/L), an elevated BUN level: [13.92 mmol/L (39 mg/dL)], and a normal creatinine level: [35.36 μmol/L (0.4 mg/dL)]. Hormonal evaluation revealed high adrenocorticotropic hormone (ACTH) 275 pmol/L (>1250 pg/mL) (normal: 0-10.12 pmol/mL) and normal luteinizing hormone (1.24 IU/L) and follicle-stimulating hormone (1.08 IU/L) levels. Baseline and corticotropin-stimulated cortisol, 17(OH) progesterone, 1.4 androstenedione and dehydroepiandrosterone sulfate (DHEA-S) levels are presented in Table 1. Baseline and stimulated levels of cortisol, 17(OH) progesterone and androstenedione were low, whereas DHEA-S levels were not as low as was expected according to the age-matched references (12). Plasma renin activity; 32.32 μg/L/hr (normal: 1.9-6.0 μg/L/h) was high, whereas aldosterone level; 0.19 nmol/L (7.1 ng/dL) (normal:0.96-8.31 nmol/L (35-300 ng/dL) was low. The karyotype of the patient was 46,XY. A diagnosis of adrenal insufficiency was made, and treatment with hydrocortisone, fludrocortisone and salt was initiated, to which the patient responded well. Although the patient was initially misdiagnosed as congenital adrenal hyperplasia (CAH) in the ICU, the high ACTH levels with insufficient adrenal steroid production associated with the patient’s clinical features suggested that the adrenal insufficiency was related to abnormal adrenal development rather than a steroid biosynthesis defect. Thus, a novel hemizygous frame shift mutation in DAX1 (c.543delA) which would cause loss of function was identified by DNA sequence analysis. DAX1 analyses of both parents were normal.

## DISCUSSION

The incidence of X-linked AHC is estimated to be between 1:140 000 and 1:200 000 (4). However, there are only two cases reported from Turkey and no reported incidence. The first of these cases was a newborn who presented with adrenal insufficiency on the seventh postnatal day and who had a hemizygous deletion of exons 1 and 2 of DAX1 (13). The second case was a male infant admitted with adrenal failure who showed a novel mutation of Q155X in DAX1 gene and who interestingly developed gonadotropin-dependent precocious puberty at the age of 9 months (14). Our patient had a hemizygous frameshift mutation in DAX1 and, to our knowledge, is the third Turkish patient reported. Frame shift mutations affect protein function severely. These types of mutations frequently cause disease.Since the initial identification of DAX1 as the gene responsible for AHC, numerous additional mutations have been discovered including deletions, alterations of splice-sites, missense mutations, nonsense mutations and frame shift mutations (4,7,11,15,16,17,18,19,20,21,22,23,24,25,26,27).

The clinical picture of AHC is variable. Approximately 60 % of the boys with DAX1 mutations have an early onset of primary adrenal failure presenting with salt wasting in the first two months of life that can be misdiagnosed as CAH (11,27,28). In 25 Chinese infant boys with primary adrenal insufficiency, DAX1 gene mutations were found in 40% (29). Other cases are reported to have a more insidious presentation - symptoms becoming obvious later in childhood and which may be triggered by a stressful incident. A newborn patient who had presented with primary adrenal failure due to missense mutation in DAX1 had an asymptomatic 8-month-old brother with the same mutation, and adrenocorticotropin stimulation test showed impaired adrenal function in the asymptomatic brother (11). This bimodal pattern of presentation may reflect normal age-related changes in mineralocorticoid secretion and sensitivity, in sodium and fluid intake, and in counterregulatory responses (15).

X-linked AHC is known to be associated with hypogonadotropic hypogonadism (HHG). Prolonged survival of these children into adulthood has shown that HHG is commonly associated with this disorder (28,30). Although the site of the deficiency within the hypothalamic-pituitary axis has not yet been pinpointed (31), the studies in two kindreds with HHG have shown that the hypothalamus as well as the pituitary and gonads are affected (26,27).

In females, heterozygous mutations in the DAX1 gene have been shown to be associated with delayed menarche, whereas homozygous mutations are related with HHG (8).

The differential diagnosis of AHC includes CAH, adrenoleukodystrophy, and exceptionally, congenital defects of the hypothalamus and pituitary (13). Careful clinical evaluation and hormonal measurements are essential for the diagnosis. In our patient, the serum levels of 17-OH progesterone and other measured steroids were low, whereas ACTH level was increased, indicating cortisol deficiency not related to 21-hydroxylase or other adrenal enzyme deficiencies. Salt-wasting crisis with high renin levels showed aldosterone deficiency. Normal male genital development indicated that testosterone synthesis was normal. Primary adrenal failure that was not associated with adrenal steroid biosynthesis defect suggested failure in adrenal gland development and led to the analysis of DAX1 gene by which the mutation was detected. Baseline and stimulated serum DHEA-S levels of our patient were not as low as expected, findings which might be due to normal production of DHEA by the testes, which can cross-react with DHEA-S (in the analysis) derived from the adrenal. It is also possible that the fetal adrenal zone, the major fetal source of DHEA and DHEA-S, had developed despite DAX1 deficiency.

The mutational analysis of DAX1 is important not only for the provision of correct genetic advice to the families but also for the appropriate management of the patients. A typical example of the unfortunate outcome of such cases is the report of a male patient who had been followed as a case of CAH until 24 years of age when he was admitted with gonadal failure and was found to have a mutation in the DAX1 gene (28). Although at present only the adrenal gland of our patient seems to be affected, his pituitary and gonadal functions will be carefully evaluated at his follow-up visits.

**Acknowledgements**

The authors thank Joseph A.Majzoub, M.D. (Chief, Division of Endocrinology, Boston Children’s Hospital, Harvard Medical School) for his comments and suggestions for the patient’s evaluation and for careful review of this manuscript.

## Figures and Tables

**Table 1:**
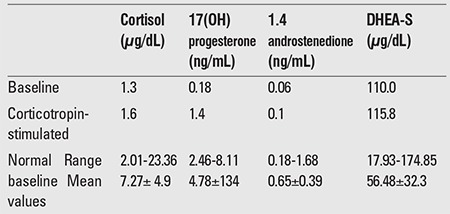
Baseline and corticotropin-stimulated levels of adrenal androgens in the patient as compared with normal baseline values (12)
